# Finding inhabited settlements and tracking vaccination progress: the application of satellite imagery analysis to guide the immunization response to confirmation of previously-undetected, ongoing endemic wild poliovirus transmission in Borno State, Nigeria

**DOI:** 10.1186/s12942-019-0175-y

**Published:** 2019-05-16

**Authors:** Jeff Higgins, Usman Adamu, Kehinde Adewara, Adeshina Aladeshawe, Aron Aregay, Inuwa Barau, Andrew Berens, Omotayo Bolu, Nina Dutton, Nnaemeka Iduma, Bryant Jones, Brian Kaplan, Sule Meleh, Melton Musa, Gatei wa Nganda, Vincent Seaman, Anupma Sud, Stephane Vouillamoz, Eric Wiesen

**Affiliations:** 1 Geospatial Research, Analysis and Services Program (GRASP), Agency for Toxic Substances and Disease Registry, Centers for Disease Control and Prevention, 4770 Buford Hwy NE, Atlanta, GA 30341 USA; 2 National Polio Emergency Operations Center, Abuja, Nigeria; 3 eHealth Africa, Maiduguri, Nigeria; 4 Bill and Melinda Gates Foundation, Seattle, USA; 5 World Health Organization, Abuja, Nigeria; 6 National Primary Health Care Development Agency, Abuja, Nigeria; 7 Global Immunization Division, Centers for Disease Control and Prevention, Atlanta, USA; 8 Solina Health, Maiduguri, Nigeria; 9 Borno State Primary Health Care Development Agency, Maiduguri, Nigeria; 10 African Field Epidemiology Network, Maiduguri, Nigeria; 11 Novel-T Sàrl, Geneva, Switzerland

## Abstract

**Background:**

Four wild polio-virus cases were reported in Borno State, Nigeria 2016, 1 year after Nigeria had been removed from the list of polio endemic countries by the World Health Organization. Resulting from Nigeria’s decade long conflict with Boko Haram, health officials had been unable to access as much as 60% of the settlements in Borno, hindering vaccination and surveillance efforts. This lack of accessibility made it difficult for the government to assess the current population distribution within Borno. This study aimed to use high resolution, visible band satellite imagery to assess the habitation of inaccessible villages in Borno State.

**Methods:**

Using high resolution (31–50 cm) imagery from DigitalGlobe, analysts evaluated the habitation status of settlements in Borno State identified by Nigeria’s Vaccination Tracking System. The analysts looked at imagery of each settlement and, using vegetation (overgrowth vs. cleared) as a proxy for human habitation, classified settlements into three categories: inhabited, partially abandoned, and abandoned. Analysts also classified the intact percentage of each settlement starting at 0% (totally destroyed since last assessment) and increasing in 25% intervals through 100% (completely intact but not expanded) up to 200+% (more than doubled in size) by looking for destroyed buildings. These assessments were then used to adjust previously established population estimates for each settlement. These new population distributions were compared to vaccination efforts to determine the number of children under 5 unreached by vaccination teams.

**Results:**

Of the 11,927 settlements assessed 3203 were assessed as abandoned (1892 of those completely destroyed), 662 as partially abandoned, and 8062 as fully inhabited as of December of 2017. Comparing the derived population estimates from the new assessments to previous assessment and the activities of vaccination teams shows that an estimated 180,155 of the 337,411 under five children who were unreached in 2016 were reached in 2017 (70.5% through vaccination efforts in previously inaccessible areas, 29.5% through displacement to accessible areas).

**Conclusions:**

This study’s methodology provides important planning and situation awareness information to health workers in Borno, Nigeria, and may serve as a model for future data gathering efforts in inaccessible regions.

## Introduction

The Global Polio Eradication Initiative (GPEI) was established in 1988 as a partnership of the World Health Organization (WHO), Rotary International, UNICEF, and the US Centers for Disease Control and Prevention, when the World Health Assembly resolved to eradicate polio by the year 2000; the Bill and Melinda Gates Foundation joined the partnership more recently. While great strides were achieved by 2000, wild poliovirus (WPV) transmission was still endemic in 24 countries [1]. By 2006 the number of endemic countries had been reduced to four [2], and by 2012, WPV transmission remained endemic in only three countries: Pakistan, Nigeria, and Afghanistan [3]. In 2015, Nigeria had not reported a WPV case for over 1 year and was removed from the WHO list of polio-endemic countries [4]. However, in 2016, four WPV cases were reported from Borno State in Nigeria [5], representing a major setback to GPEI.

For the past 10 years, northeastern states in Nigeria, and in particular Borno, have been plagued by violence committed by the Boko Haram terrorist insurgency. According to the WHO, the group is responsible for killing over 20,000 people since 2009 and the displacement of approximately two million people [6]. In 2012 and 2013, the group began occupying villages and taking over large swaths of Borno State, including entire Local Government Areas (LGAs). In 2014 and 2015, 50–60% of settlements in Borno State were inaccessible to the government of Nigeria and therefore no polio eradication activities could be implemented in those areas. With massive displacement of the population, it was not known how many people remained in the Boko Haram-controlled areas. Infrastructure including health facilities and cellular network towers were shut-down or destroyed precluding communication with remaining residents. In addition, displaced people fleeing the Boko Haram areas had not been able to move around freely and therefore were not aware of the status of neighboring communities.

Although there were WPV cases reported in Borno in 2014, the WPV detected in 2016 was most closely genetically linked to WPV detected in Borno State in 2011 [5], indicating prolonged undetected transmission of that lineage for approximately 5 years. Lack of access for immunization activities or acute flaccid paralysis surveillance in insurgency-held areas allowed ongoing endemic WPV transmission to remain undetected [5]. After continued endemic WPV transmission was confirmed in 2016, there was a crucial need to better understand the location and size of the populations living in the Boko Haram-controlled areas of Borno to identify the extent of gaps in immunity and surveillance.

High resolution satellite imagery has been used to locate and estimate populations in past studies, including the vaccination tracking system (VTS) project which determined settlement locations and modeled settlement populations to assist the polio program (Nigeria’s government and GPEI partners) in tracking mass vaccination efforts in northern Nigeria [7]. However, these data were produced before much of the displacement began in Borno, limiting their value in assessing current populations. Other studies have also successfully used satellite imagery to identify structures which have been damaged or destroyed in conflict [8], or to estimate the population size of displaced persons in camp-like settings [9]. However, a method for determining the habitation status of an intact settlement in an inaccessible area has not been demonstrated.

We present a project that developed such a method, which was combined with damage assessments to update pre-displacement population estimates from the VTS to aid the polio program and other humanitarian agencies.

## Methods

### Background and data sources

To assist the polio program in Borno in determining which areas were still inhabited and estimating the population of children under 5 years of age (the target age for oral polio vaccination; referred to henceforth as “u5” children) in those areas, we developed a methodology to manually assess inhabitancy at the settlement level using high resolution satellite imagery (commercial satellite imagery from DigitalGlobe’s WorldView-1, WorldView-2, WorldView-3, and GeoEye-1 satellites). This imagery, consisting of panchromatic and pan-sharpened color imagery at resolutions ranging from 31 to 50 cm [10], was used to manually review each settlement in the conflict-affected areas of Borno over multiple points in time. The review included an assessment of the status of the structures in the villages and an assessment of evidence of recent human habitation based on patterns of vegetation in heavily-used areas of the settlements such as paths and compound courtyards.

Nigeria’s VTS was the main source of reference data. The VTS has settlement-level data that includes settlement locations, extents, and modeled population estimates of various demographics, including u5 children [11, 12]. Settlement location data were primarily generated through automatic feature extraction, the process by which a machine-learning algorithm scans a geographically referenced satellite image or aerial photo and identifies, classifies, and clusters human structures into functional settlement boundaries. From there, field data collection was used to add settlement names and administrative affiliations [state, LGA, and ward (subdivisions of LGAs)]. Microcensus data from representative neighborhood types were factored into the model to generate settlement-level population estimates by age group [13]. For Borno State, the original VTS data were based on the status of settlements between 2012 and 2015. Therefore, the VTS data provided a pre-displacement baseline to be adjusted based on observed changes using our methodology.

### Assessing settlement habitation status

An initial satellite imagery exploration of Marte LGA in Borno, an area affected severely by the displacement crisis with no accessible settlements, provided the basis for the assessment methodology. Current imagery at the time (September 2016) was compared with imagery from earlier in 2016 and previous years. Pre-conflict imagery was analyzed to understand what inhabited and undamaged settlements looked like at various points throughout the year.

**Fig. 1:**
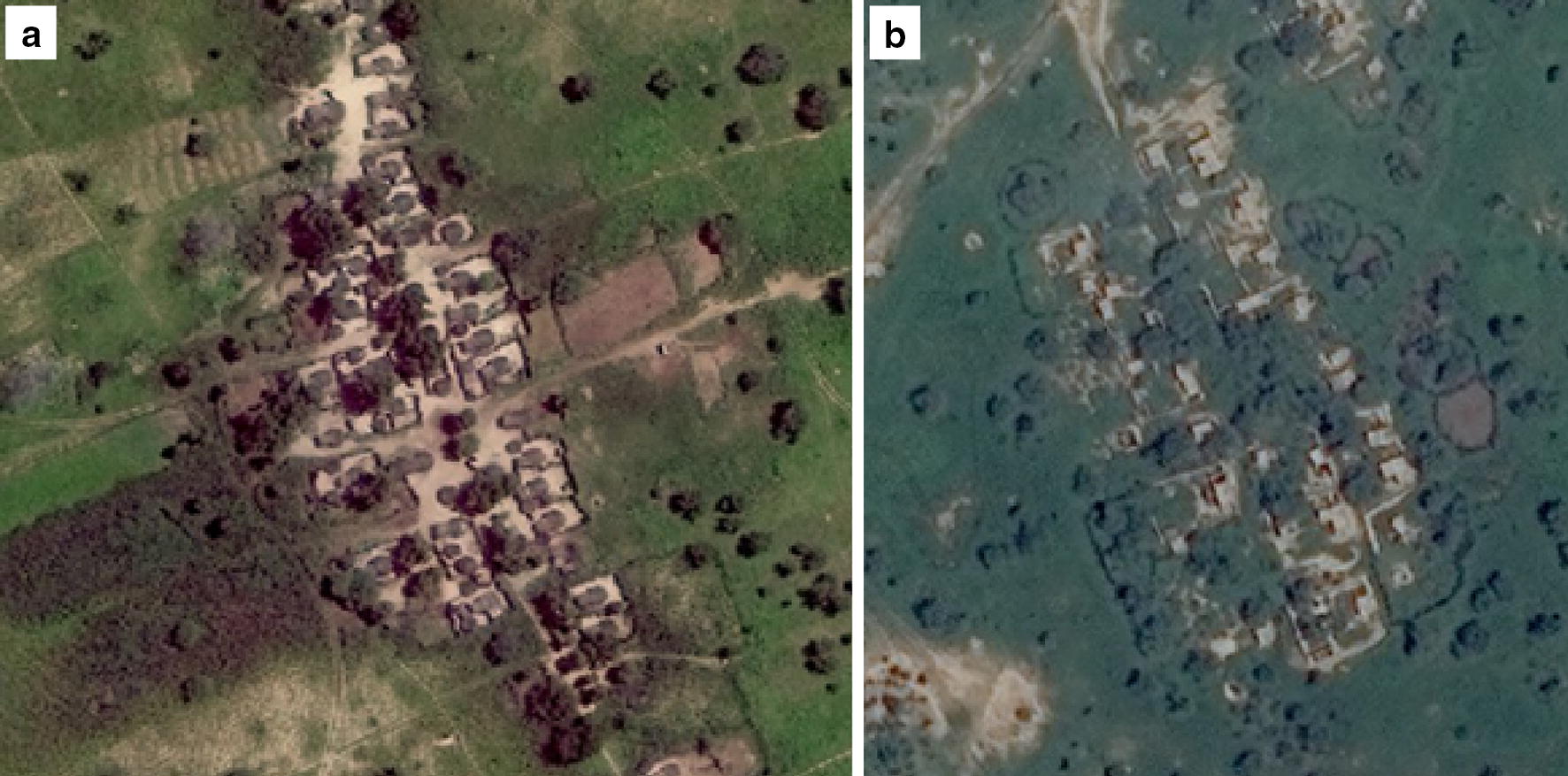
A settlement with evidence of human habitation in Gubio LGA (30 August 2016, WorldView03) (**a**), an uninhabited settlement of similar size in neighboring Mobbar LGA (29 July 2016, WorldView02) (**b**). Both color images were taken at the end of the rainy season in 2016 (image copyright DigitalGlobe)

Borno consists mostly of semi-arid steppe or sub-tropical deserts. Annual precipitation is low and concentrated between June and August [14, 15]. Vegetation grows quickly after the start of the rains, covering bare ground with thick grass [16]. Because of this rapid and distinct growing season, it is possible to assess the growth of vegetation over paths and compounds within settlements—visible in < 1 m resolution imagery—to determine whether they have been inhabited over the course of the rainy season. In inhabited places, human activity causes paths and the areas within compound walls to remain free of vegetation (Fig. 1a), similar to what could be seen in pre-conflict imagery. In uninhabited places, previously cleared areas are covered with vegetation by the end of the rainy season (Fig. 1b).

The coincidental timing of the initial exploration of the imagery, occurring just after the rainy season ended in September 2016, drew our attention to the noticeable differences between inhabited and uninhabited settlements at that time of year. From that point onward, it was observed that the overgrowth of vegetation from the most recent rainy season becomes dry and less obvious, but remains visible until the start of the following rainy season. No new overgrowth occurs in settlements abandoned during the dry season until the following rainy season. This means that there is an annual refresh rate for identifying newly-abandoned places. However, if an overgrown settlement becomes re-inhabited during the dry season, new paths and cleared areas through the dry vegetation can become evident.

From this evidence, we categorized data in an assessment attribute called *Inhabitancy*. *Inhabitancy* was divided into 3 categories: *fully inhabited*, *partially abandoned*, and *abandoned*. Settlements were assessed as *fully inhabited* if the paths through the settlement, paths between structures, and areas within compound walls were free of vegetation overgrowth. Settlements were assessed as *abandoned* if all of the areas within compound walls and paths between structures were overgrown. Occasionally, a well-traveled road or path may cut through the center of a settlement. This thru-path remaining clear of vegetation was not considered sufficient evidence of habitation if all other paths and courtyards were overgrown. In other cases where there was evidence of habitation in some parts of a settlement, but evidence of abandonment in others, the settlement was assessed as *partially abandoned*.

### Assessing settlement damage/destruction

**Fig. 2:**
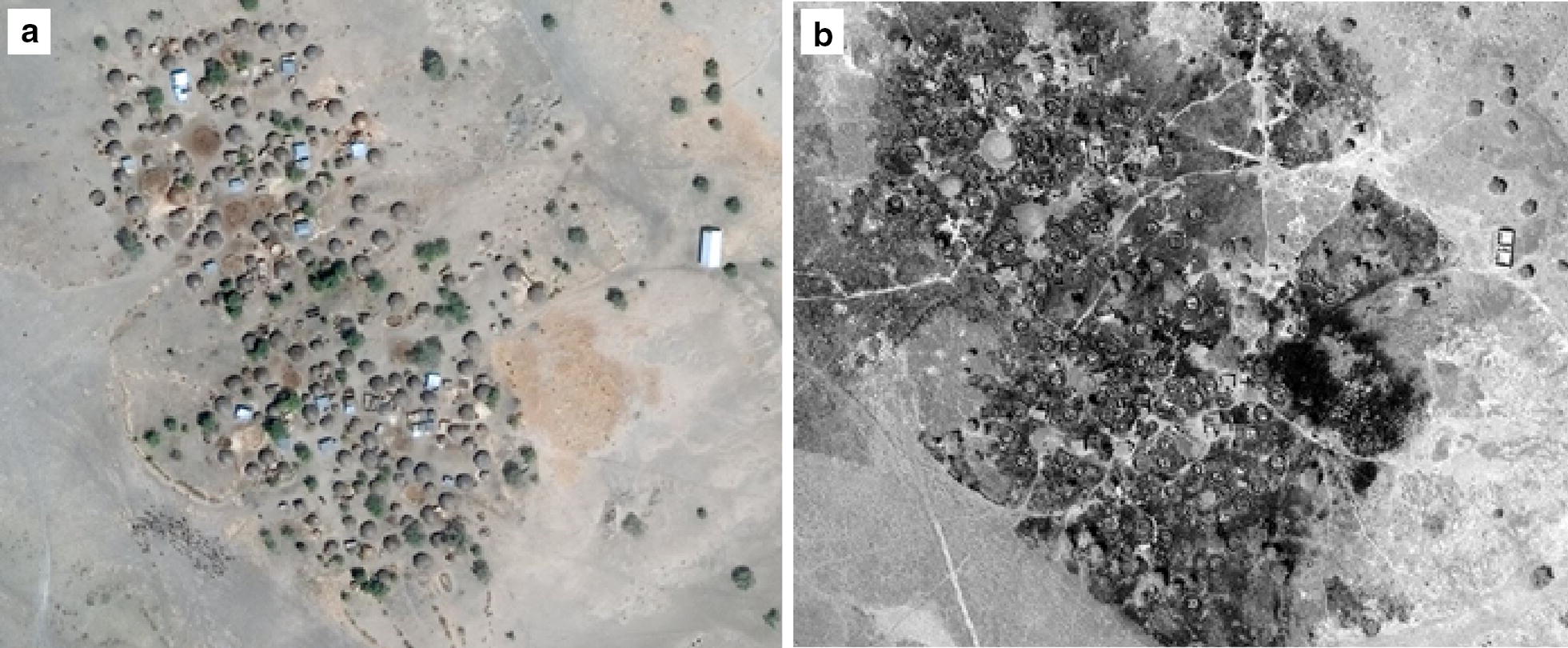
Before (**a**) (26 February 2016, WorldView02) and after (**b**) (29 January 2017, WorldView01) images of a settlement destroyed by fire in Kala Balge LGA (image copyright DigitalGlobe)

Destroyed buildings can be distinguished using high resolution satellite images by comparing images of the same structures from before and after potential destruction has taken place. In places with fire damage, it is common to see “skeletons” of buildings where the interior walls are visible after the roofs have been burnt away (Fig. 2). In other places, destruction was more comprehensive and only vague footprints of buildings remain. Destroyed settlements are evident in any subsequent image and are not subject to the same annual refresh limitation as abandoned settlements.

We categorized data on settlement damage and destruction in the assessment attribute called the *percentage of intact structures*. The *percentage of intact structures* was divided into 9 categories, ranging from 0% to 200%+ by 25% increments, allowing for the indication of reduction, growth, or no change in settlement size from earlier images. This classification scheme was designed so that reviewers could rapidly make an approximate visual assessment by selecting a category rather than calculating a precise percentage (additional attributes collected were the date of the image used for the assessment, an indication of the subjective quality of the data, and optional notes).

### Adjusting population estimates

Each settlement in the project’s area of interest (the 22 of 27 LGAs in Borno containing inaccessible areas) was assessed simultaneously for both the *percentage of intact structures* (based on manual review of level of destruction) and *level of inhabitancy* (based on manual review of vegetation patterns). These assessments were then joined based on geographic location to the VTS settlement-level data. From there, the populations of settlements classified as *abandoned* were adjusted to zero, while *fully inhabited* and *partially abandoned* settlements had their population adjusted by multiplying by the *percentage of intact structures*.^1^

### Vaccination reach

Since the immunization response to identification of ongoing WPV transmission began in Borno in 2016, all vaccination efforts, including house-to-house teams and special interventions, have been tracked using Android phones with global positioning system (GPS) applications as part of VTS. For the purposes of this study, we defined the terms *reached* and *unreached* as vaccination status classifications that could be applied to settlements and their estimated populations. The classification *reached* indicates settlements that have GPS evidence of at least one visit by tracked vaccination teams during working hours of a vaccination campaign or intervention since the start of the response in August 2016, or over a specified period. *Unreached* is the classification for settlements which do not have evidence of visitation by vaccination teams, but still show evidence of habitation. Together this binary classification scheme of *reached* and *unreached* has been termed *vaccination reach*.

**Fig. 3:**
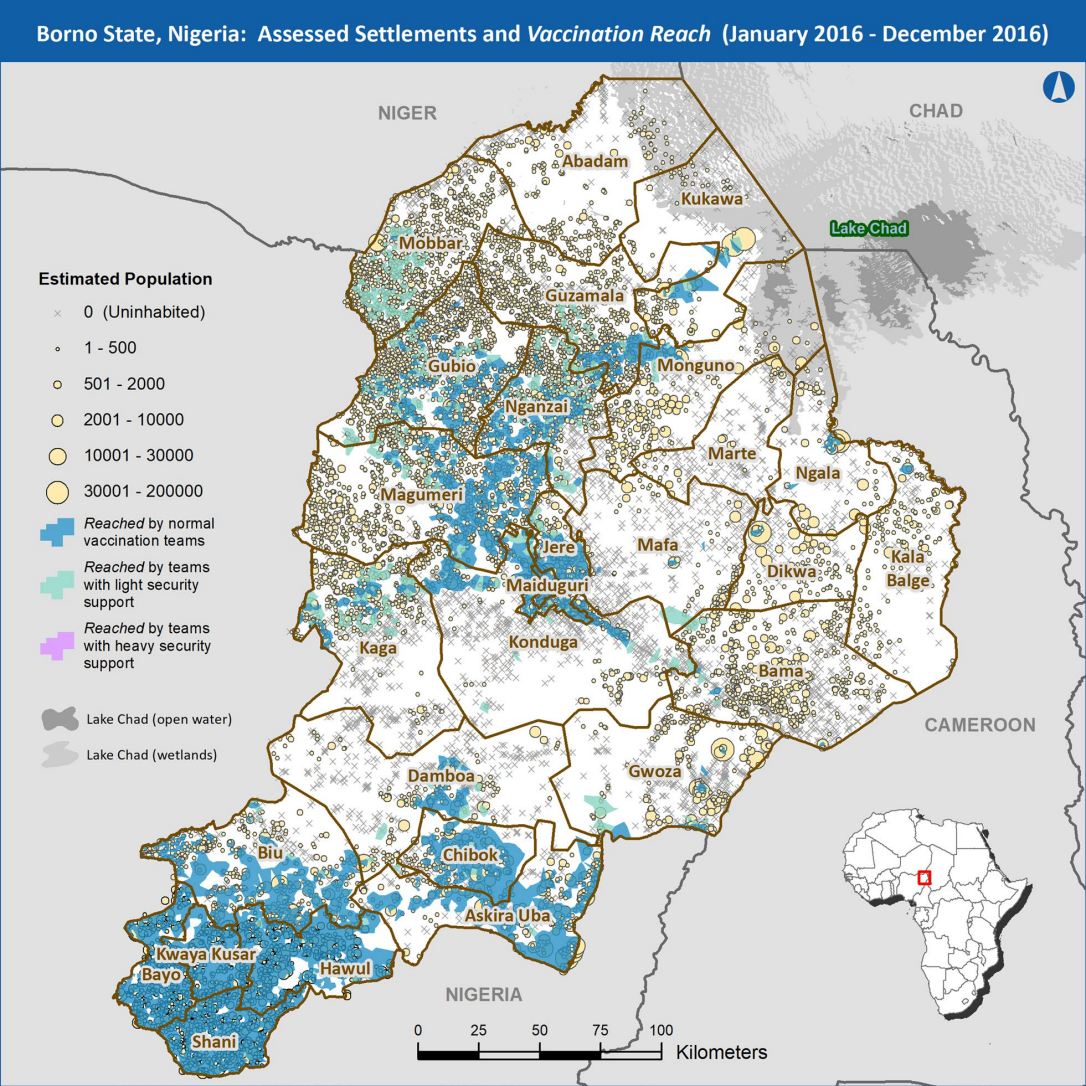
Inhabited/abandoned settlements and *vaccination reach*, December 2016

**Fig. 4:**
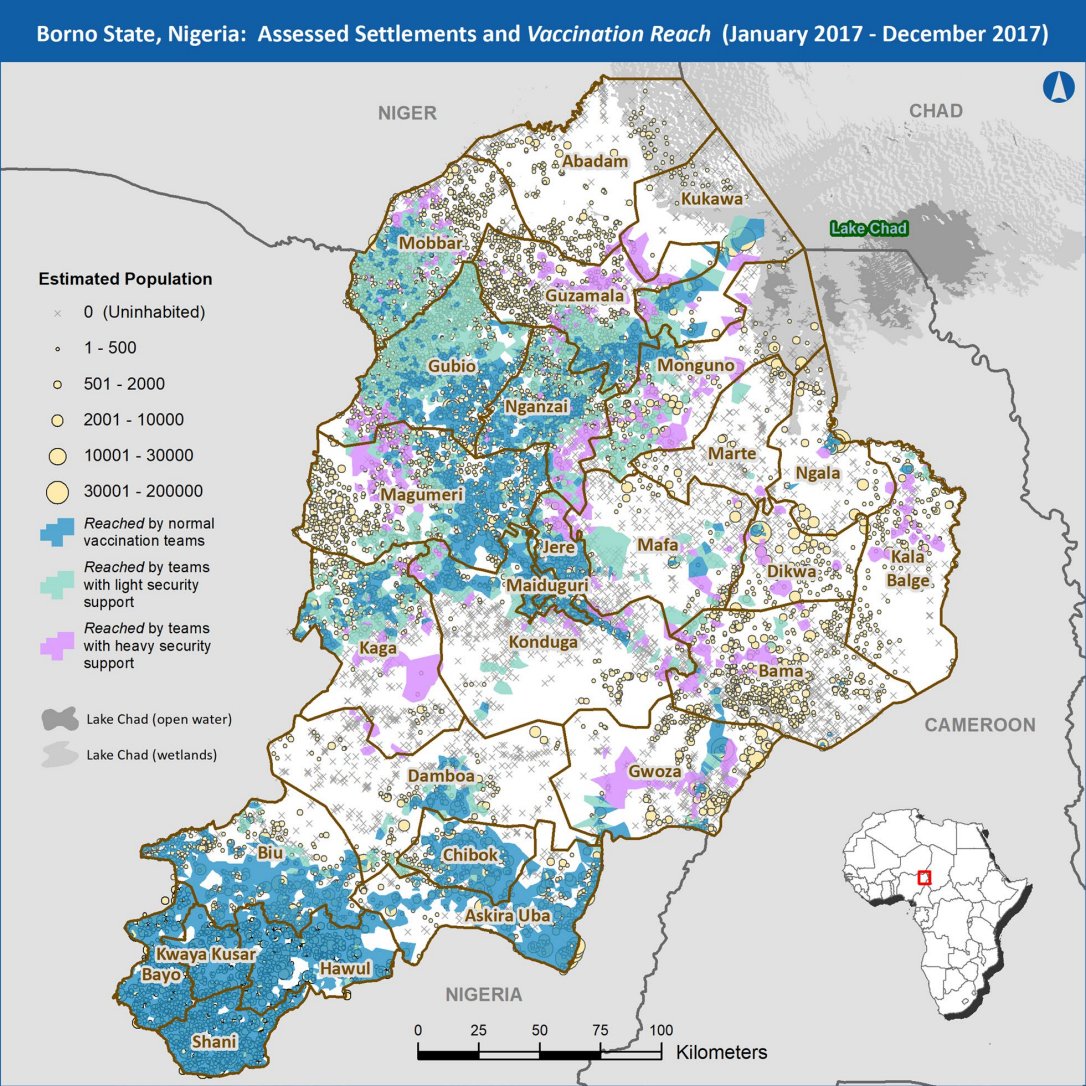
Inhabited/abandoned settlements and *vaccination reach*, December 2017

The adjusted settlement-level population estimates for *unreached* settlements were aggregated to generate a rough estimate of the total number of *unreached* u5 children at the ward, LGA, and state levels. Maps were also created with a representation of *vaccination reach* overlaid onto settlements, symbolized by their adjusted population and status for 2016 and 2017 (Figs. 3, 4, respectively). This visualization highlights both program progress and settlements that remain *unreached*.

## Results

### Settlement habitation results

As of December 2017, all of the settlements identified and represented in the geographic information system (GIS) database (n = 11,927) for the 22 of 27 Borno LGAs included in this study were assessed (in 5 LGAs, all areas were accessible and so excluded). 1892 settlements (15.9%) were found to be completely destroyed (*abandoned* with 0% of *structures intact*), 1311 settlements (11.0%) were found to be *abandoned* while at least partially intact (*intact structures 25% and greater*), 662 settlements (5.6%) were assessed as *partially abandoned*, and 8062 settlements (67.6%) were adjudged to be *fully inhabited* as shown in Table 1.

**Table 1:** Settlement habitation reach

Settlement status assessment	Number of settlements	% of total settlements
Abandoned	3203	26.9
0% intact (destroyed)	1892	15.9
25% intact or greater	1311	11.0
Partially abandoned	662	5.6
Fully inhabited	8062	67.6

### Vaccination reach results

Using this study’s methodology for the population in all of Borno’s 27 LGAs, 337,411 out a total of 1,109,312 u5 children were classified as *unreached* at the end of 2016. At the end of 2017, the estimate for *unreached* u5 children reduced to 157,256. This number represents an improvement of 180,155 previously *unreached* u5 children now classified as *reached* at least once by polio vaccination in 2017 compared to the previous year’s estimate (Table 2).

**Table 2:** Unreached U5 children overall

Year	*Unreached* u5 children	% of total u5 population
2016	337,411	30.4
2017	157,256	14.2
2016–2017 ∆	180,155	

There are two complementary reasons why more children came to be *reached* in 2017. The first was increased geographic coverage by polio vaccination teams moving into previously unvisited areas through multiple intervention strategies. The second was displacement of people from inaccessible areas to accessible areas (such as IDP camps) where they could receive vaccination more readily. Breaking down the estimated improvement of 180,155 additional *reached* u5 children into these two categories shows 126,975 of those u5 children (70.5%) were reclassified as *reached* due to increased geographic coverage by polio vaccination interventions. 53,180 u5 children (29.5%) were reclassified as *reached* by being displaced into an accessible area, assuming that the former inhabitants of deserted places were displaced to IDP camps or host communities in nearby accessible areas.

The estimate of u5 children considered *reached* due to increased geographic coverage by polio vaccination teams can be further broken down by the three main types of intervention employed (Table 3): (1) regular house-to-house vaccination teams operate in areas accessible to civilians without security support. This category accounted for 40,726 of the 126,975 u5 children (32.1%) considered *reached* due to increased geographic coverage by vaccination teams in 2017; (2) Reaching Every Settlement initiative teams operate in “partially accessible” areas where civilians can travel, but light security support is needed. This category accounted for 62,170 of the 126,975 u5 children (49.0%); and (3) Reaching Inaccessible Children initiative teams are specialized immunization teams led by security personnel which operate in areas that are inaccessible to civilians. This category accounted for 24,079 of the 126,975 u5 children (19.0%).

**Table 3:** Unreached U5 children improvement by reason

Reason	u5 children reclassified as *reached* at the end of 2017	% of total *reach* improvement
*Reached* by house to house teams	40,726	22.6
*Reached* by Reaching Every Settlement teams	62,170	34.5
*Reached* by Reaching Inaccessible Children teams	24,079	13.4
Displaced to accessible areas	53,180	29.5
Total	180,155	100.0

It is possible that a settlement may have been visited by multiple interventions throughout the year as the security situation in that area improved or deteriorated. In these cases, settlements and their populations were counted according to the least intensive intervention that visited them during the year and not in any other category. The maps in Figs. 3 and 4 show the inhabited and abandoned settlements of Borno by adjusted population as of 31 December 2017. Each map also shows a representation of the geographic areas classified as *reached*, broken down by the three different vaccination strategies described above at the end of 2016 (Fig. 3) and at the end of 2017 (Fig. 4). As vaccination with oral poliovirus vaccine requires multiple doses to optimize the proportion of children who develop immunity, the number of vaccination intervention visits to a given area is being documented to estimate a proxy for number of doses given to the u5 population.

### Validation

The polio program in Borno confirmed that our assessments provided a reasonably accurate situation awareness of the conditions on the ground. In January 2017, the GIS analysts leading this project met in Borno with local teams to validate the settlement assessment methodology and share initial findings. The feedback shared from the Borno teams anecdotally confirmed a number of settlement assessments and provided preliminary support for the model’s validity. In October 2017, an analysis was conducted comparing the settlement status assessment from the satellite imagery analysis with a field assessment of settlement status conducted by security personnel as part of their support for vaccination in inaccessible areas between May and September of 2017. The satellite-based assessments agreed with the field data from security personnel in 1375 (84.3%) of 1631 settlements compared (Table 4). In Table 4, a TRUE value for the settlement status data indicates a settlement which was assessed per our methodology as *fully inhabited* or *partially abandoned*, while a FALSE value indicates a settlement assessed as *abandoned*. For the security personnel field data, a TRUE value indicates that inhabitants were actually found in that place, while a FALSE value indicates there were actually no inhabitants found.

**Table 4:** Sensitivity and specificity of settlement status assessments

	Security personnel field data		
Settlement status data	True	False	
True	1064	186	PPV = 85.1%
False	70	311	NPV = 81.6%
	Sensitivity = 93.8%	Specificity = 62.6%	Accuracy = 84.3%

*PPV* positive predictive value, *NPV* negative predictive value

## Discussion

Prior to the discovery of prolonged WPV circulation in August 2016, it was assumed that the inaccessible areas of Borno State were depopulated to the extent that WPV would not continue to circulate. Once that assumption was proven false, the polio program was faced with the possibility that without evidence to the contrary, all inaccessible settlements could be inhabited and thus must be visited by vaccination teams to interrupt transmission. This is a costly assumption in terms of resources required to visit all settlements and the danger faced by vaccinators. The method described for this project provided the program with information that could supplant those assumptions. Our results showed large, contiguous inhabited areas such as the western LGAs of Magumeri, Gubio, and Mobbar, while revealing other areas that were depopulated to varying degrees such as the eastern LGAs of Bama, Dikwa, and Kala Balge (see Figs. 3, 4). There was substantial agreement between the satellite imagery analysis data and the preliminary field data collected by security personnel in inaccessible areas. The agreement was sufficiently high to indicate that the habitation data from this study has actionable implications for vaccination intervention planning in Borno. Settlements that were destroyed or abandoned could be de-prioritized from vaccination planning while settlements with signs of habitation could be prioritized. The effect of prioritization was evident when comparing the first two rounds of the Reaching Inaccessible Children vaccination intervention. In the first round, planning did not prioritize settlements based on our results and 64% of inaccessible settlements visited were found to be uninhabited. In the second round, places assessed in this project as *fully inhabited* or *partially abandoned* began to be prioritized and only 32% of settlements visited were found to be uninhabited.

Our data are subject to several limitations. First, there is a limited degree of precision possible in manual reviews of satellite images. Because the available satellite imagery was not of high enough resolution to view humans directly, estimates were based on indirect evidence of habitation such as the presence of intact structures and paths free of vegetation overgrowth. With manual image interpretation, there is always potential for human error as well. This was mitigated by using a team approach to assessment, where analysts were rotated through different parts of the state and difficult assessments were brought before a group for discussion. Second, waiting for new vegetation overgrowth after the annual rainy season to assess inhabitancy means that the gap between the true situation on the ground and what is discernable in imagery widens throughout the year until the next rainy season. In addition, the ability to update the status of some priority areas is limited by the availability of recent imagery, as obtaining satellite images over priority areas is not always timely or feasible. Third, the method for estimating populations relied on the modeled settlement-level population estimates in the VTS as a baseline input; any inaccuracy or error in the baseline estimates would be carried through this analysis. Finally, the determination of whether or not a settlement should be classified as *reached* was based on the number of GPS tracking points that intersected with the boundaries of the settlement. The concept of *vaccination reach* based on geographic VTS tracking data is at best a proxy for the actual occurrence of vaccination. If an inhabited place was visited while teams were being tracked, but complete vaccination did not take place, it may lead to an erroneous conclusion that virtually all of the u5 children in that settlement were vaccinated. Also, changes in habitation density (i.e. due to IDPs moving into currently inhabited structures) are not discernable by our method and we were not able to account for increased populations per settlement in the absence of an increase in the number of structures. In the future, incorporating IDP count data from the Displacement Tracking Matrix produced by the International Organization for Migration can potentially mitigate this issue [17].

We assumed that the former inhabitants of deserted places were displaced to IDP camps or host communities in accessible areas. It is possible, however, that some of these children moved to other inaccessible areas, leading to an underestimation of the number of *unreached* children. While numbers of *reached* children are mentioned in this study, these numbers are not intended to be a replacement for tally data of children who have received vaccination, nor is this method designed to replace any other monitoring measures in place in the polio program such as lot quality assurance sampling (LQAS). Rather, this method provided situational awareness data, such as the locations of inhabited settlements in the insurgency-held areas where no other methods of data collection were available. For this reason, the estimates of *unreached* u5 children and the changes to the *unreached* numbers are emphasized instead of *reached* totals, which risk being misconstrued as a direct count of vaccinated children.

This methodology could be of use in other areas experiencing both displacement and inaccessibility, given sufficient time and resources. Geo-referenced settlement locations and access to high resolution satellite imagery are necessary prerequisites. If imagery covering the area of interest is already available, one trained analyst or a small team could rapidly assess a small area of approximately 1000 settlements in a week. Larger areas would likely require an increase in time or personnel. In the course of this project, the pace of satellite imagery collection over areas of interest, rather than personnel, was the biggest constraint on the pace of assessments. As a result, it took our team of four analysts several months to cover all 11,927 settlements.

Future work could focus on the automation of this methodology. The development of automated methods for conducting the imagery analysis could reduce the total time needed to assess an area and make this method easier to implement in resource-constrained settings. A follow-up study is underway to automate the assessment process via deep learning with convolutional neural networks, using the human-generated settlement assessments from this study as training data.

## Conclusion

Our method of using satellite imagery to estimate populations and locations of settlements in Borno State, Nigeria provided vital information and situational awareness to the polio program in Nigeria. This method was also used by the greater humanitarian response for planning activities and forecasting humanitarian needs in Borno State. The situational awareness provided by this study enabled the polio program to gain support from security forces in vaccinating populations in inaccessible areas of Borno in 2017. Intense efforts were made to access children in prioritized areas leading to an increase in approximately 86,000 u5 children that were considered *reached* by polio vaccination in inaccessible areas with the assistance of security personnel in 2017. A further 41,000 were considered *reached* by regular vaccination teams in areas where it became safe to operate after improvements in security.

As of December 2017, our methodology estimated that approximately 157,000 u5 children were living in settlements still inaccessible to the polio program in Borno State. This is a reduction of approximately 180,000 children from the estimate for December 2016. Without this knowledge, the program would have no way of estimating its progress toward ending WPV transmission. This analysis strongly suggests that increased geographic coverage by vaccination activities, as well as effective acute flaccid paralysis surveillance, is needed in inaccessible areas of Borno before WPV transmission can be considered interrupted. Notably, the security-supported vaccination initiatives mentioned above have continued in 2018. Continuing to update the settlement status assessments regularly (especially after each year’s rainy season) can provide usable data until the end of the polio immunization response and humanitarian crisis in Borno. The settlement status analysis is still ongoing and being updated as new imagery becomes available.

In summary, the development and application of the novel approach for assessing settlement habitation status in inaccessible areas using satellite imagery and the integration of those data with existing datasets and systems as described in this article is an important advancement in the use of GIS technology for addressing public health priorities among inaccessible populations. This method has the potential to improve planning and implementation of public health interventions for at-risk populations trapped in conflict-affected regions around the world.

## Data Availability

The data that support the findings of this study are available from DigitalGlobe and the Vaccination Tracking System of Nigeria but restrictions apply to the availability of these data, which were used under license for the current study, and so are not publicly available.

## Footnotes

1 While the *partially abandoned* category provided valuable situational awareness information to the polio program, it was treated the same as *fully inhabited* when determining population adjustments to avoid having to estimate the *percent abandoned*, which may be more highly prone to error.
